# ^18^F-AV-1451 in Parkinson’s Disease with and without dementia and in Dementia with Lewy Bodies

**DOI:** 10.1038/s41598-018-23041-x

**Published:** 2018-03-16

**Authors:** Ruben Smith, Michael Schöll, Elisabet Londos, Tomas Ohlsson, Oskar Hansson

**Affiliations:** 1 Department of Neurology, Skåne University Hospital, Lund, Sweden; 2 Clinical Memory Research Unit, Department of Clinical Sciences, Lund University, Malmö, Sweden; 3 Wallenberg Centre for Molecular and Translational Medicine and the Department of Psychiatry and Neurochemistry, University of Gothenburg, Gothenburg, Sweden; 4 Memory Clinic, Skåne University Hospital, Malmö, Sweden; 5 Department of Radiation Physics, Skåne University Hospital, Lund, Sweden

## Abstract

Mixed pathologies of α-synuclein, β-amyloid and tau are relatively common in Parkinson’s disease (PD) and Dementia with Lewy Bodies (DLB). We therefore wanted to study the retention patterns of ^18^F-AV-1451 in PD, PD-dementia (PDD), and DLB. To do this 44 healthy controls, 11 non-demented patients with PD, 18 patients with PDD, and six patients with DLB underwent MRI and ^18^F-AV-1451 PET scanning and cognitive testing. We found that parietal ^18^F-AV-1451 retention was increased in patients with DLB compared to controls and PD patients, while ^18^F-AV-1451 uptake was reduced in the substantia nigra in PDD. Increased parietal ^18^F-AV-1451 PET uptake was associated with impaired performance on verbal fluency tests, and the decreased uptake in the substantia nigra correlated with worse motor function. We found no effect of the monoamine oxidase B inhibitor rasagiline on ^18^F-AV-1451 binding. In conclusion DLB patients have increased parietal ^18^F-AV-1451 uptake. Increased parietal tau is associated with executive impairment in patients with synucleinopathies, while decreased uptake in the substantia nigra is associated with parkinsonism. Further, our data indicate that ^18^F-AV-1451 does not significantly bind to MAO-B *in vivo*.

## Introduction

A large proportion of patients with Parkinson’s Disease (PD) eventually develop cognitive impairment and dementia^1^. In patients with Dementia with Lewy Bodies (DLB), cognitive decline should by definition precede or occur within one year of the appearance of motor symptoms^2^. Neuropathologically, the two diseases exhibit Lewy bodies in the neocortex, in limbic structures and in the brainstem, as well as loss of midbrain dopaminergic cells^3–5^. Further, coincidental AD pathology is also relatively common in PD and common in DLB^5,6^. ^18^F-AV-1451 has been demonstrated to bind to paired helical filament (PHF)-tau aggregates^7,8^. We hypothesised that cortical retention of ^18^F-AV-1451 would be increased in the subjects affected by dementia compared to the non-demented controls and sought to investigate the usefulness of this PET-tracer in the diagnostic work-up for these different disorders.

In addition to tau aggregates, AV-1451 has been shown to bind to neuromelanin in the substantia nigra^7,9,10^. This binding was reduced in patients with PD and PD with mild cognitive impairment compared to controls^11^. To study whether the levels are reduced in PDD and DLB, and if they correlate with motor function, we assessed ^18^F-AV-1451 retention in substantia nigra in our study participants.

## Study participants and Methods

### Participants

Informed written consent was obtained from all patients before inclusion in the study. All procedures were approved by the Regional ethics committee at Lund University, the Radiation protection committee at Skåne University Hospital, and the Swedish Medical Products Agency. All experiments were performed in accordance with relevant guidelines and regulations. The study participants were recruited from the Neurology and Memory clinics at Skåne University Hospital, Sweden as part of the ongoing Swedish BioFINDER study (www.biofinder.se). Controls within the BioFINDER-study were enrolled from the prospective Malmö Diet and Cancer study cohort^12^. We included 29 patients with PD diagnosed according to the National Institute of Neurological Disorders and Stroke (NINDS) diagnostic criteria for PD^13^, out of which 18 patients fulfilled criteria for PD with dementia^14^; six patients with DLB according to the McKeith *et al*.-criteria^15^; and 44 neurologically healthy, age-matched controls.

Lumbar puncture was performed and cerebrospinal fluid (CSF) collected and handled as previously described^16^. The patients were assessed with Hoehn and Yahr staging^17^, UPDRS part III^18^, and by neurologic examination through a physician experienced in movement disorders. Patients were assessed on medication. Further, all study participants were tested cognitively by a registered nurse on an occasion separate to the imaging. Cognitive tests used were: Mini-Mental State Exam^19^, letter S- and animal fluency^20^, A Quick Test for cognitive speed (AQT)^21^, and the wordlist delayed recall part of the Alzheimer’s Disease Assessment Scale-cognitive Subscale (ADAS-cog)^22^. Amyloid positivity was defined as either ^18^F-Flutemetamol composite score >1.42^23^ or CSF β-Amyloid_42_ <550 pg/ml (clinical reference cut-off).

### Image acquisition and processing

#### MRI

T1-weighted magnetization-prepared rapid gradient echo (MPRAGE) and Fluid-attenuated inversion recovery (FLAIR) images were acquired for all patients on a 3 T Siemens Skyra scanner (Siemens Medical Solutions, Erlangen, Germany) and processed along with the PET images using an in-house developed pipeline, described previously^8^ (for sequence details please see Supplementary Methods).

#### PET

The radiosynthesis procedure, radiochemical purity, and scanning methods for ^18^F-AV-1451 have been described in detail previously^24^. Subjects underwent a simplified protocol including one ^18^F-AV-1451 scan 80–100 min (4 × 300 s frames) post injection on a GE Discovery 690 PET scanner (General Electric Medical Systems, Milwaukee, WI, USA). PET data was processed using an in-house developed pipeline described earlier^8^, using FreeSurfer 5.3 segmentation of the MRI, and standardized uptake value ratio (SUVR) calculation using the inferior cerebellar grey matter as reference region. SUVR values derived from the FreeSurfer-segmented regions of interest (ROIs) were pooled into larger composite ROIs (frontal; medial and lateral parietal; medial, inferior and lateral temporal; and occipital cortex) for further analysis. The exact composition of these ROIs is described in the Supplementary Methods. Ligand retention in the basal ganglia was analyzed separately using Pmod 3.711 (PMOD technologies llc; Zürich, Switzerland) to replicate the method used in^9^. Input images were the SUVR images in MNI space derived from the above-mentioned imaging pipeline. Template ROIs for the substantia nigra as well as the globus pallidus were drawn on an MRI in atlas space, and the individual PET images were then coregistered to the MRI with the fusion tool. Volume of distribution (V_d_) was calculated as previously described^9^.

### Statistics

**Figure 1:**
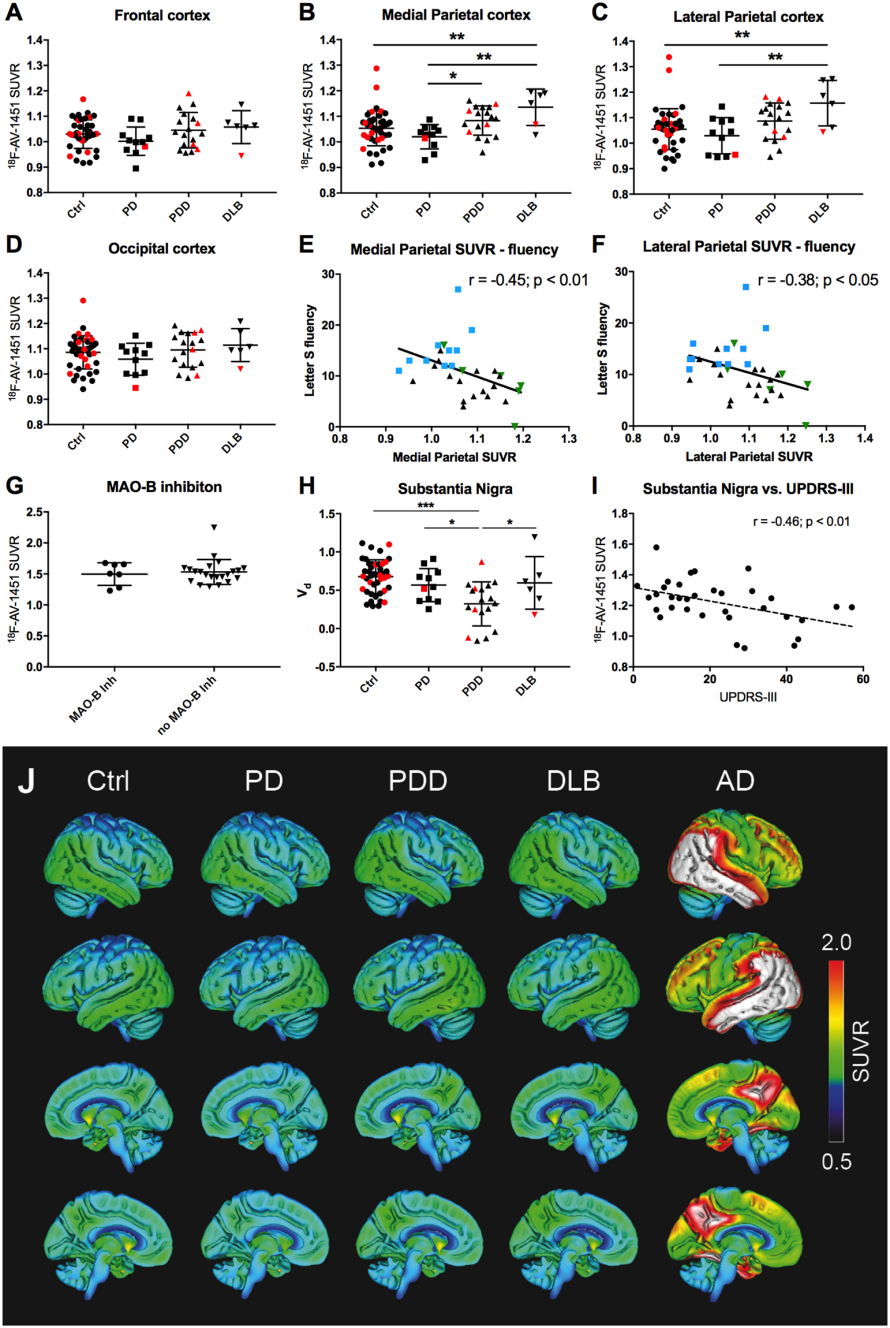
^18^F-AV-1451 retention in cortical and subcortical regions. ^18^F-AV-1451 SUVRs in the frontal cortex (**A**), medial parietal cortex (**B**), lateral parietal cortex (**C**) and occipital cortex (**D**). Amyloid positive subjects are represented by red shapes and amyloid negative subjects by black shapes. Correlations of medial (**E**) and lateral (**F**) parietal cortex SUVRs to Letter S-fluency scores. PD = blue squares; PDD = black triangles; DLB = green inverted triangles. (**G**) The effect of MAO-B inhibition on SUVRs in the globus pallidus. (**H**) ^18^F-AV-1451 SUVRs in the substantia nigra. (**I**) Correlation of ^18^F-AV-1451 SUVRs in the substantia nigra and the Unified Parkinson’s Disease Rating Scale (UPDRS) motor score. (**J**) Lateral and medial view of average SUVR images of the diagnostic groups studied. The rightmost column represents an average SUVR image of typical AD-cases (n = 41) that have been included merely for visual comparison. White color in the AD images represents saturation of the SUVR-scale (i.e. SUVR > 2). Ctrl - controls; DLB - Dementia with Lewy Bodies; Inh - inhibitor; MAO-B - monoamine oxidase B; PD - Parkinson’s Disease; PDD - Parkinson’s Disease with dementia; V_d_ – Volume of distribution. *p < 0.05, **p < 0.01, ***p < 0.001.

Statistical analyses were performed using IBM SPSS statistics for Macintosh, version 23. For comparisons between groups ANCOVA was used, adjusted for age. Chi-square tests were used to compare non-continuous variables (gender and amyloid positivity). T-tests were used to compare AV-1451 uptake in relation to MAO-B inhibitor intake. Correlations in Fig. 1 and Supplementary Figure 2 were performed using Pearson correlations. Finally, linear regression models adjusted for age and clinical diagnosis were performed. Statistical significance was assumed at p < 0.05.

AV-1451 uptake patterns were further compared between respective patient groups and control subjects employing a voxel-wise two-sample t-test as implemented in SPM12 (http://www.fil.ion.ucl.ac.uk/spm) adjusted for age. The results were thresholded at p < 0.01, uncorrected for multiple comparisons regarding the limited sample size. All AV-1451 SUVR images had been transformed into common Montreal Neurological Institute (MNI) space by using transformation measures from warping the co-registered MRI scans into the 2 mm MNI152 MRI template and smoothed with an 8 mm full width at half maximum (FWHM) kernel prior to the analysis.

## Results

### Participants

Study participants were recruited from the Neurology and Memory clinics at Skåne University Hospital, Sweden. We found no differences in gender, proportion of amyloid positive subjects, or education between the groups. PD patients were slightly younger than the other participants. As expected patients with PDD or DLB performed worse on cognitive testing compared to the other groups. All clinical characteristics are presented in Table 1.

**Table 1:** Clinical characteristics of study participants.

	Controls (C) (n = 44)	PD (n = 11)	PDD (n = 18)	DLB (n = 6)	Significant differences
Disease duration (yr)	N/A	5.1 ± 4.0	9.0 ± 4.1	5.5 ± 2.2	PD < PDD*
Age	76 ± 5.3	67 ± 5.5	73 ± 7.0	71 ± 6.3	PD < PDD*, C > PD***, C > PDD*
Gender (M/F)	21/23	8/3	12/6	3/3	n.s.
Aβ-status (positive/negative)	13/31	1/8^§^	4/12^§^	1/3^§^	n.s.
UPDRS III	N/A	11 ± 8	27 ± 13	26 ± 21	PD < PDD**
MMSE	29 ± 1	28 ± 2	22 ± 4	22 ± 7	C > PDD***, C > DLB***, PD > PDD***, PD > DLB***
Education	11.7 ± 3.8	11.9 ± 2.8	12.3 ± 4.4	10.5 ± 2.8	n.s.
AQT (CS)	64.7 ± 12.5	68.5 ± 15.3	149.5 ± 73.5	89.0 ± 30.3	C < PDD***, PD < PDD***, PDD > DLB**
Word list delayed recall (ADAS)	2.3 ± 1.8	1.8 ± 1.4	6.5 ± 2.9	7.7 ± 2.9	C < PDD***, C < DLB***, PD < PDD***, PD < DLB***
Letter S-fluency	16 ± 7	15 ± 5	9 ± 3	9 ± 5	C > PDD***, C > DLB**, PD > DLB*, PD > PDD**
Animal fluency	24 ± 6	22 ± 6	11 ± 6	12 ± 8	C > PDD***, C > DLB***, PD > PDD***, PD > DLB**

Aβ - Amyloid beta; ADAS - Alzheimer’s Disease Assessment Scale; AQT (CS) - A Quick Test for cognitive speed (Colour and shape); N/A - Not applicable; MMSE - Mini-mental State Exam; UPDRS III – Unified Parkinson’s Disease Rating Scale, item III (motor score). n.s. – not significant. *p < 0.05, **p < 0.01, ***p < 0.001. ^§^Data on amyloid status are missing on two subjects in the PD, PDD and DLB groups.

### Cortical retention of AV-1451

We observed increased ^18^F-AV-1451 retention in the medial and lateral parietal lobes of patients with DLB compared to controls (p < 0.01) and non-demented patients with PD (p < 0.01) (Fig. 1, Supplementary Figure 1). In the medial areas of the parietal lobe we found increased uptake in PDD compared to PD (p < 0.05; Fig. 1). In the inferior temporal lobes a slightly lower retention of AV-1451 was seen in the PD-group compared to controls and PDD, but no other differences were observed (Supplementary Figure 2). No differences were observed between groups in other brain regions (p > 0.05; Fig. 1). Furthermore, we found no effect of amyloid positivity on AV-1451 SUVR in subjects with synucleinopathies (p > 0.05).

Next, we studied the associations between cognitive function and ^18^F-AV-1451 retention in the cortical areas of the included patients with synucleinopathies (PD, PDD and DLB). Significant correlations were found for Letter S fluency scores and tracer retention in both the medial and lateral parietal regions (r = −0.45; p < 0.01 and r = −0.38; p < 0.05 respectively. Figure 1E,F). Similar results were obtained for animal fluency (r = −0.49; p < 0.01 and r = −0.42; p < 0.05). All effects remained statistically significant after correcting for age and diagnostic groups. The data were not corrected for multiple comparisons. No correlations were seen with cortical retention of AV-1451 and MMSE.

Since it had been reported that an MAO-B inhibitor reduced uptake of the tau PET-tracer ^18^F-THK5351^25^, and more recently not affected retention of ^18^F-AV-1451^26^, we compared ^18^F-AV-1451 retention in our study participants treated with the MAO-B inhibitor rasagiline (n = 7) with those without this medication (n = 22). We observed no differences in the cortical or subcortical retention of ^18^F-AV-1451 between these two groups (p > 0.05; Fig. 1G).

### Retention in the substantia nigra and the globus pallidus

Next, we evaluated ^18^F-AV-1451 retention in the substantia nigra. We found no difference between controls and non-demented PD participants, however, a significant decrease could be observed when comparing PDD to all other groups (Fig. 1H). The decrease in SUVR in the substantia nigra in subjects with PD/PDD/DLB correlated with an increase in the UPDRS motor scale (r = −0.46; p < 0.01; Fig. 1I). This remained statistically significant after correcting for age and diagnostic groups.

We have previously reported off-target AV-1451 binding that increases with age in the globus pallidus in controls and in patients with progressive supranuclear palsy^27^. In this study we observed no difference between groups in the globus pallidus after adjusting for age (data not shown). However, as expected, we found a strong correlation between ^18^F-AV-1451 retention in the globus pallidus and age (Supplementary Figure 2).

## Discussion

We report slightly, yet significantly increased retention of ^18^F-AV-1451 in the parietal cortex of patients with DLB when compared to non-demented PD patients and controls. The increase in parietal AV-1451 uptake in DLB was clearly lower than retention levels typically seen in Alzheimer’s disease. While our results are largely in agreement with two recently published articles showing subtle increases in AV-1451 uptake in parietal regions in DLB cases, these studies did also report increased uptake in inferior and lateral temporal lobes^28^ as well as in large parts of the occipital lobes^29^, which we could not confirm in the current study. Possibly this could be attributed to the selection of controls for the studies. In the previously published studies^28,29^, controls were selected to be amyloid negative, whereas in the present study controls were included irrespective of amyloid status. Our results however, did not change substantially when excluding the amyloid positive controls or amyloid positive subjects over all. Another possible explanation to the differences between the studies may be the limited number of DLB cases in the present study. Even though we found increased retention of AV-1451 in the parietal lobes of DLB patients, the overlap between the diagnostic groups were substantial and the clinical usefulness of the tracer in differential diagnosis of PD and DLB is likely limited. Further, the retention of the ligand is low in DLB, and since neuropathological correlates of AV-1451 retention and tau pathology is currently lacking in DLB, we cannot exclude that the signal detected may be related to general neurodegeneration and not to tau pathology per se.

Verbal fluency tests have been suggested to be sensitive to detect cognitive deficits in DLB^5^. In the present study, we found statistically significant associations between AV-1451 uptake in the parietal cortex and both letter S fluency and animal fluency, but not with memory function (word list delayed recall) or global cognition (MMSE). Kantarci and colleagues did not find an association between cognitive function and AV-1451 uptake in DLB. They did, however, not explore associations with fluency or other tests reflecting cognitive speed or executive function^29^. Gomperts *et al*. report correlations to MMSE with retention of AV-1451 in the inferior temporal gyrus^28^, a finding that we could not replicate in the current study. The cortical retention of AV-1451 in DLB and PDD is low, especially comparing to the levels of retention seen in AD (Fig. 1j). The correlations between AV-1451 and cognitive decline are sparse in our PDD and DLB cases. Taken together, we believe that other pathologies than tau are responsible for the larger part of the cognitive decline seen in the PDD and DLB subjects.

In the present study, we found no increase in AV-1451 retention in the cortex of PD patients irrespective of cognitive status compared to controls. Similarly, Hansen *et al*. did not observe increased cortical retention of AV-1451 in a relatively large sample of non-demented PD patients^11^. Comparable results were also reported from another study in non-demented PD patients^30^. Moreover, decreased AV-1451 retention has previously been reported for the substantia nigra in PD and PD with mild cognitive impairment, potentially reflecting the loss of neuromelanin containing cells^9,30^. We found similar results in our study, observing decreased AV-1451 retention in the substantia nigra of PDD patients compared to controls. Furthermore, we could for the first time show that the decreased AV-1451 uptake in the substantia nigra correlated significantly with more pronounced parkinsonism according to UPDRS-III. No difference in the AV-1451 uptake in substantia nigra was observed between controls and PD subjects, this could possibly be due to too small group sizes. The decrease in substantia nigra signal is also affected by disease duration (Suppl Fig. 2c). Since PD subjects have a shorter disease duration than PDD subjects this may in part explain the absence of a difference between controls and the non-demented PD group.

The MAO-B inhibitor selegiline has been demonstrated to block binding of the tau PET tracer THK5351^25^. However, selegiline or rasagiline, was recently reported not to affect the retention of AV-1451^26^. A small number (n = 7) of the participants in this study were on treatment with the MAO-B inhibitor rasagiline (none on selegiline). We did not observe any difference in the AV-1451 retention in the cerebral cortex, the globus pallidus, or the substantia nigra in these subjects when compared to participants not taking the drug. The data on MAO-B inhibition presented in Fig. 1 depicts SUVR-values. Similar findings were obtained using SUVs to account for potential MAO-B expression in the cerebellum. The number of patients on rasagiline in this comparison was low, and our data acquired in a cross-sectional manner, which renders any conclusion preliminary. Nonetheless, the results are in line with another recently published study^26^ and these result potentially suggests lower affinity of AV-1451 to MAO-B than THK5351.

In previous publications, we and others have reported age-related off-target binding in the globus pallidus in control subjects and patients with Progressive Supranuclear Palsy (PSP)^27,31–34^. Here, we found no effect of age on the retention of AV-1451 in any brain region except for the globus pallidus where age and pallidal AV-1451 SUVRs were highly correlated (Suppl Fig. 2). This could potentially reflect an age-dependent iron accumulation^31^.

Limitations: One limitation of the study is the limited number of cases with DLB included. The results for DLB subjects should be interpreted with this in mind. Further, due to the exploratory nature of the study no correction for multiple comparisons was applied to the data. The main results of SUVRs in different brain regions would survive a correction for multiple comparisons except PD vs PDD in the medial parietal cortex and PD vs PDD and PDD vs DLB in the substantia nigra. The correlations of cognitive fluency tests to AV-1451 retention do not survive correction for multiple comparisons.

In conclusion, we report increased SUVRs in the parietal lobes of patients with DLB compared to healthy control and non-demented PD participants. In addition, parietal AV-1451 retention correlated with worse performance on verbal fluency tests in patients with synucleinopathies. Decreased substantia nigra AV-1451 uptake was associated with more pronounced parkinsonism. Finally, the MAO-B inhibitor rasagiline did not affect retention of AV-1451.

## Electronic supplementary material

Supplementary information
