# Supplementary material for: 18F-AV-1451 in Parkinson’s Disease with and without dementia and in Dementia with Lewy Bodies
Source: Sci Rep. 2018 Mar 16;8:4717. doi: 10.1038/s41598-018-23041-x (PMC5856779; doi:10.1038/s41598-018-23041-x)
Supplement: Supplementary file 1 — Supplementary information [file 41598_2018_23041_MOESM1_ESM.pdf]

Supplementary information:

^{18}F-AV-1451 in Parkinson's Disease with and without dementia and in Dementia with Lewy Bodies.

Ruben Smith, MD, PhD; Michael Schöll, PhD; Elisabet Londos, MD, PhD; Tomas Ohlsson, PhD and Oskar Hansson, MD, PhD.

MRI sequences

- MPRAGE: 1 x 1 x 1 mm^3, 176 slices, TR/TE/TI 1900/2.64/900 ms
- FLAIR: 0.7 x 0.7 x 5 mm^3, 20 % distance, 23 slices, TR/TE/TI 9000/81/2500 ms

Composite ROIs

The composite ROIs used for analyses in the manuscript were composed of the following Desikan-Killiany Atlas regions from FreeSurfer 5.3:

Frontal ROI: Bilateral superior frontal, rostral middle frontal, caudal middle frontal pars opercularis, pars triangularis, pars orbitalis, lateral orbitofrontal, medial orbitofrontal and frontal poles.

Medial parietal ROI: Bilateral precuneus, posterior cingulate and isthmus cingulate.

Lateral prietal ROI: Bilateral superior parietal, inferior parietal and supramarginal.

Inferior temporal ROI: Bilateral inferior temporal and temporal poles.

Medial temporal ROI: Bilateral hippocampus, parahippocampus, entorhinal and fusiform.

Lateral temporal ROI: Bilateral banks of the superior temporal sulcus (bankssts), middle temporal, superior and transverse temporal.

Occipital ROI: Bilateral cuneus, lateral occipital, lingual and pericalcarine.

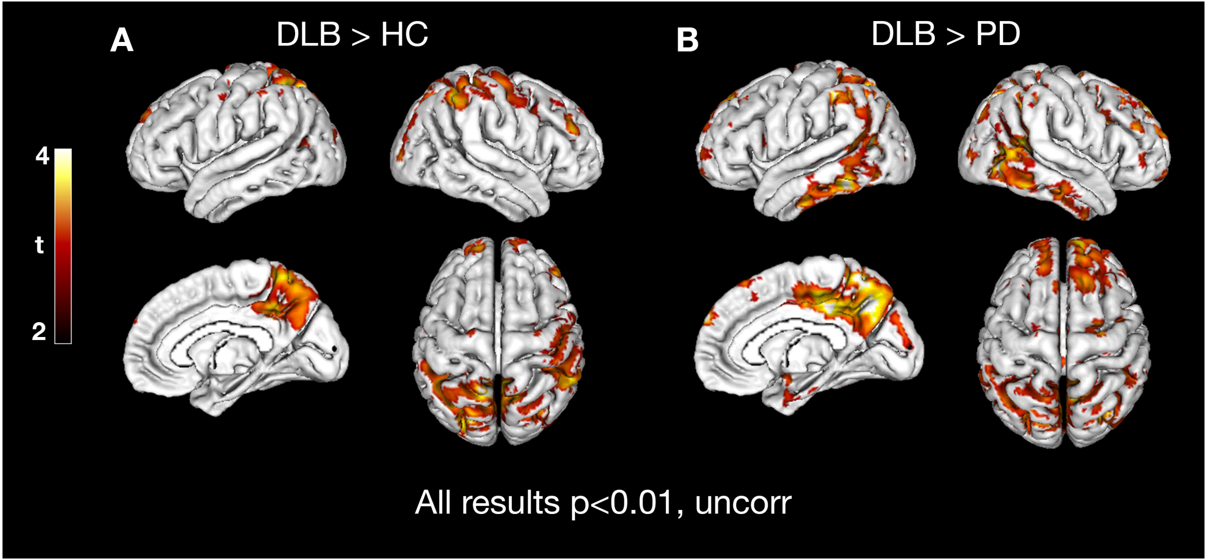

Supplementary Figure 1. Voxel-wise comparisons of DLB and healthy controls (A) and DLB and PD (B) subjects, thresholded at p<0.01, uncorrected for multiple comparisons.

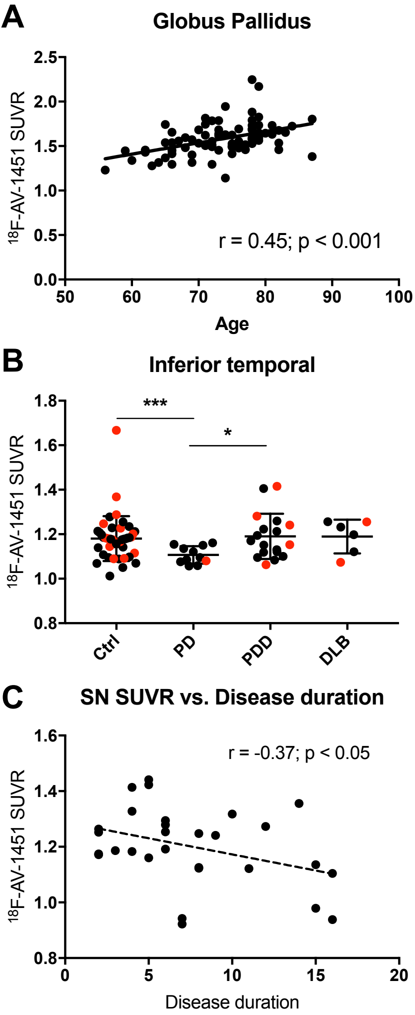

Supplementary Figure 2. A) Correlation of Globus pallidus SUVR to participant age. B) SUVR-values in the inferior temporal lobe. Red dots represent amyloid positive subjects. * $p < 0.05$; *** $p < 0.001$. C) Substantia nigra retention versus disease duration in subjects with Parkinson's disease.
